# Dynamic monocyte chemoattractant protein-1 level as predictors of perceived pain during first and second phacoemulsification eye surgeries in patients with bilateral cataract

**DOI:** 10.1186/s12886-021-01880-z

**Published:** 2021-03-12

**Authors:** Feng Zhang, Jin-Hua Wang, Mei-Sheng Zhao

**Affiliations:** 1 Department of Ophthalmology, The Second Clinical Medical College, Jing Zhou Central Hospital, Yangtze University, Hubei 434020 Jing Zhou, China; 2 Department of Cataract, Ophthalmic Center, The Second Affiliated Hospital of Jilin University, Jilin 130041 Chang Chun, China

**Keywords:** Bilateral cataract surgery, MCP-1, CCL2, Pain, Predictor

## Abstract

**Background:**

The purpose of the study was to investigate whether dynamic monocyte chemoattractant protein-1 (MCP-1) level might be as predictors of perceived pain during the first and second phacoemulsification eye surgeries in patients with bilateral cataract.

**Methods:**

Consecutive bilateral cataract patients undergoing bilateral sequential phacoemulsification were retrospectively enrolled. Patients’ preoperative anxiety score and intraoperative pain score were registered. Aqueous humor samples were obtained during surgery. MCP-1 level in the aqueous humor was measured by enzyme linked immunosorbent assay (Elisa). Patients were assigned to seven subgroups based on the interval between first-eye and second-eye cataract surgery. Comparisons were performed for a subjective sensation and MCP-1 levels among different subgroups.

**Results:**

pain score during second-eye surgery was significantly higher than during first-eye surgery. Whereas there was no statistical difference in anxiety score between both surgeries. Result from subgroups comparison showed that the visual analog scale (VAS) pain score was statistically greater in 1-group and 6-group during the second eye surgery. Anxiety score did not statistically differ in subgroups. Additionally, the second-eye MCP-1 level was significantly higher at week 1and 6 intervals. Preoperative MCP-1 level was positively correlated with perceiving pain score during both surgeries.

**Conclusions:**

MCP-1 level in aqueous humor significantly correlated with perceived pain during cataract surgery. Dynamic MCP-1 level could function as predictors of perceived pain during the first and second phacoemulsification eye surgeries in patients with bilateral cataract, which might support clinicians in treatment optimization and clinical decision-making.

## Background

Cataract is one of the most common and serious eye diseases leading to blindness and severe visual impairment especially in patients aged 50 years or older worldwide [1–3]. Since the 1990s, cataract surgery has progressed to the modern technique of phacoemulsification that involves a small corneal incision and the implantation of an intralobular foldable lens (IOL). This surgical procedure no longer requires complete akinesia, thus, encouraging the use of less invasive anesthetic modalities [4]. In the last decade, peribulbar, retrobulbar, and sub-Tenon’s anesthesia were the most popular techniques used during cataract surgery. However, some complications of these methods may also be observed. Nowadays, different local anesthesia (topical and intracameral) is the more commonly used [5–7]. Phacoemulsification with IOL under topical anesthesia is widely performed to treat cataract, as it has several advantages such as avoidance of postoperative ptosis or diplopia, immediate visual recovery, decreased patients’ anxiety, shorter operative time, improved intraoperative patient cooperation, and better safety [8, 9]. It is estimated that more than 5 million cataract procedures are performed annually in China [1]. Consecutive operation at intervals might be an ideal approach for treating patients with bilateral cataracts [10].

As an invasive procedure, cataract surgery might cause disruption of the blood-aqueous humor barrier, thereby resulting in production of prostaglandin in aqueous humor. Those might be associated with postoperative complications such as pain of a contralateral eye, anterior segment inflammation and macular edema especially in patients with bilateral cataracts [8, 11, 12]. Research during the last two decades revealed that patient might experience more painful sensation during second eye surgery, even though both procedures were performed by a same clinician and under same condition [13–15]. Besides, the interval between surgeries might also be associated with patients’ perceived pain [16]. Although some studies have examined the objectively physiological mechanism, it still lacks of dynamic indicator after first-eye surgery might function as predictors of perceived pain during the second eye surgery.

Inflammation is known to be closely associated with the development of pain [17, 18]. Cytokine and chemokine-mediated inflammation has been confirmed to play an important role in the pathogenesis of pain [19]. CC chemokine receptor 2 (CCR2) is one of 19 members of the chemokine receptor subfamily of human class A G-protein-coupled receptors [20]. CCR2 and its ligands are involved in numerous inflammatory and pain caused by a variety of diseases. Several cytokines and chemokines that attract the migration of leukocytes related to inflammation have been shown to be involved in the initiation of pain [21]. Among those cytokines and chemokines, monocyte chemoattractant protein-1 (MCP-1), known as CCL2, has been found universally increased in the nervous system in different models of pain [22]. By blocking MCP-1 and/or its cognate receptor CCR2 as well as in CCR2 knockout mice, attenuation of pain following nerve injury is also observed [23]. What is more, MCP-1in aqueous humor is found to be significantly increased in the fellow eye after first-eye surgery [24–26]. By dynamic monitoring MCP-1 level between first-eye and second-eye surgery, we might be able to identify possible objectively physiological mechanism and provide an optimal timing for second-eye surgery [16]. We hypothesized that dynamic MCP-1 level might be predictors of perceived pain during the first and second phacoemulsification eye surgeries in patients with bilateral cataract.

## Materials and methods

### Patients

The study recruited consecutive bilateral cataract patients undergoing bilateral sequential phacoemulsification with IOL implantation under topical anesthesia from department of Cataract, Ophthalmic center, the second affiliated hospital of Jilin university between May, 2016 and October, 2016. All patients’ sequential second-eye operation was conducted within 6 weeks after the first-eye surgery. First-eye surgery was carried out with a higher-grade cataract or otherwise poorer vision. The exclusion criteria were as follow: (1) patients with tumors or coronary heart disease who are taking oral nonsteroidal drugs ; (2) patients with a history of ocular trauma or surgery; (3) poor compliance or involuntary movement to cataract surgery under topical anesthesia; (4) patients with baseline eye pain including glaucoma or high intraocular pressure; (5) patients taking pain relief medications or therapy; (6) patients with complicated cataract. Finally, 141 patients remained and were analysed in the present study. Approval of protocol was obtained from the Ethical Committee of the second hospital of Jilin University.Written informed consent was signed from every eligible patient and their relatives. The methods were carried out according to every guideline and regulation. Record related to patient was anonymous and de-identified prior to analysis. The study was conducted in accordance with the Declaration of Helsinki.

### Surgical procedure and specimen collection

Routinely topical anesthesia using minims proparacaine hydrochloride was performed at the beginning of the operation. Then an eye speculum was used to keep the eye open and the eye was centered under an operating microscope. The conjunctive sac was washed with povidone-iodine and ample amount of normal saline in turn. After those procedures, about 100-200ul aqueous humor samples from the anterior chamber via the transparent anterior angular membrane above were obtained by 1ml injection syringe during first-eye and second eye surgery, and they were immediately stored at -80. Conventional hydrodissection, chopping, nucleus rotation, and phacoemulsification were conducted. Then an optimal IOL was implanted by a dedicated injector. Finally, the incision was hydrated with balanced salt solution and verified for water tightness after the aspiration of any residual viscoelastic material.

### Preoperative anxiety and perioperative pain evaluation

Preoperative anxiety was assessed when a patient was in the waiting room before surgery using a validated simplified State-Trait Anxiety Inventory (STAI; 6 questions) and a visual analog scale (VAS) for anxiety. VAS anxiety is presented as a numbered line ranging from 0 (no anxiety) to 10 (unbearable anxiety). Perioperative pain was immediately evaluated after surgery via a VAS for pain, which was presented as a numbered line ranging from 0 (no pain) to 10 (unbearable pain). Preoperative anxiety and perioperative pain evaluation were performed by two trained investigators, respectively.

### Data collection

Baseline data including age at surgery, gender and interval time between surgeries was obtained from medical records. Preoperative examinations related to eye including vision, intraocular pressure, axial length, and nuclear grading were also recorded. Besides, detail surgical procedures also were retrieved from surgical or nursing records.

### Measurement of MCP-1 level in aqueous humor sample

The level of MCP-1 in aqueous humor sample was measured using a commercial enzyme linked immunosorbent assay (ELISA) kit according to the instructions of the manufacturer (Dakewe, Shenzhen, China).

### Statistical analysis

Quantitative data were presented as the median (range), while categorical data were expressed as the frequency and percentage of the total group. The chi-square test was used to determine any significant difference between categorical variables. Based on their distribution, continuous variables were compared using the Wilcoxon’s rank-sum test or Student’s t-test. Linear regression analysis was used to explore significant correlations in continuous data with linear relationships. Statistical analysis was performed using PASW Statistics 23.0 software (SPSS Inc., Chicago, IL, US). Two-sided *P* value < 0.05 was considered to be statistically significant.

## Results

### Baseline characteristics

Baseline characteristics of 141 consecutive bilateral cataract patients (67 males and 74 females; median age 69 years; range, 44–86 years) undergone consecutive cataract surgeries were summarized in Table 1. Among those patients, 55 patients (39.0 %) and 32 patients (22.7 %) had concomitant hypertension or diabetes mellitus, respectively. The median axial length, intraocular pressure, nuclear grading, and interval time of both surgeries were 23mm (range 20-27mm), 15mmHg (range 10–20 mmHg), 4 (range 2–5), and 25 days (1–49 days), respectively. In terms of cataract classification of second eye, a majority of patients were classified into the nuclear type.

**Table 1:** Baseline characteristics of patients

Patients Characteristics	Number of patients (percentage)
Gender	
Female	74 (52.5 %)
Male	67 (47.5 %)
Age at surgery (median, range) (years)	69 (44–86)
Site of second surgery (Left)	74 (52.5 %)
Hypertension (present)	55 (39.0 %)
Diabetes mellitus (present)	32 (22.7 %)
Classification of cataracts	
Nuclear	86 (61.0 %)
Cortical	15 (10.7 %)
Posterior subcapsular	11 (7.87 %)
Mixed	29 (20.6 %)
Axial length (median, range) (mm)	23 (20–27)
Intraocular pressure (median, range) (mmHg)	15 (10–20)
Nuclear grading	4 (2–5)
Interval time of surgery (median, range) (days)	25 (1–49)

### Comparison of patients between first‐surgery and second‐surgery

Comparisons between first-surgery and second-surgery were recorded in Table 2. There was no significant difference between two groups in terms of baseline characteristics including the second surgical site, cataract classification, nuclear grading, axial length, and intraocular pressure. The median operation room time and phacoemulsification time were 18.5 min (range 13–29 min) and 3 min (range 2–6 min) for the first-surgery and 19 min (range 13–28 min) and 3 min (range 2–6 min) for the second-surgery. The differences were not statistically significant (*P*_operation room time_ = 0.769 and *P*_phacoemulsification time_ = 0.754).

**Table 2:** Comparison of characteristics between first and second cataract surgery

Parameter	First-eye surgery	Second-eye surgery	*P* value
Site surgery (Left vs. Right)	67/74	74/67	0.404^a^
Classification of cataract			0.860^a^
Nuclear	79	86	
Cortical	16	15	
Posterior subcapsular	13	11	
Mixed	33	29	
Axial length	23 (19–27)	23 (20–27)	0.564^b^
Intraocular pressure	15 (10–21)	15 (10–20)	0.642^b^
Nuclear grading	4 (1–6)	4 (2–5)	0.325^b^
Median operating room time (min)	18.5 (13–29)	19 (13–28)	0.769^b^
Median phacoemulsification time (min)	3 (2–6)	3 (2–6)	0.754^b^
MCP-1 (pg/ml)	2773(1027–3888)	3357 (2271–4989)	*< 0.001*^*b*^
Median value of anxiety (range)			
STAI	9 (6–24)	8 (6–27)	0.101^b^
VAS anxiety	2 (0–8)	2 (0–8)	0.114^b^
Median VAS pain (range)	1 (0–7)	2 (0–7)	*< 0.001*^*b*^

The *P* value of less than 0.050 was considered to indicate a significant difference

*HR* heart rate, *STAI* State–Trait Anxiety Inventory, *VAS* visual analog scale, *MCP-1* monocyte chemoattractant protein-1

Note: ^a^X^2^ test; ^b^Wilcoxon test

**Fig. 1:**
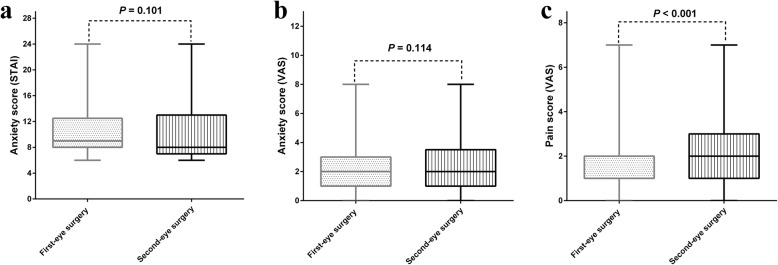
Comparison of anxiety and pain score between the first and second phacoemulsification cataract surgery. The bar in the box indicates the median. The lower and upper bars indicate the range (minimum- maximum). **a** STAI anxiety score, **b** VAS anxiety score, **c** VAS pain score

Although the median anxiety scores were relatively and slightly lower in patients who underwent second-eye surgery than those in patients who underwent first-eye surgery, there was no significant difference between the two groups (*P*_STAI_ = 0.101 and *P*_VAS_ = 0.114) (Fig. 1a-b). However, VAS pain score during second-eye surgery was significantly greater (median: 2, range: 0–7) than those during first-eye surgery (median: 2, range: 0–7) (shown in Fig. 1c) (*P* < 0.001).

### Comparison of patients in different subgroups

All the recruited patients were subdivided into seven subgroups based on the intervals between first-eye and second-eye cataract surgery. The exact number of patients in each subgroup was 22, 19, 20, 21, 20, 20, and 19, respectively. Each subgroup was named as group 1, group 2, group 3, group 4, group 5, group 6, and group 7 based on the time order.

Comparative data of different subgroups were shown in Table 3. Statistical analysis demonstrated no significant difference was found in gender, age at surgery, site of a second-surgery, cataract classification, concomitant diseases, and objective indexes including nuclear grading, axial length, and intraocular pressure among subgroups. Additionally, there was no significant difference in operation time between the two groups.

**Table 3:** Comparison of baseline characteristics among subgroups

Patients Characteristics	1-group	2-group	3-group	4-group	5-group	6-group	7-group	*P* value
Gender (Male/Female)	11/11	7/12	12/8	10/11	9/11	9/11	9/10	0.893^a^
Age at surgery (years)	70.50 (44–80)	68 (46–86)	68 (50–82)	70 (46–84)	70 (51–81)	68.5 (49–86)	71 (44–78)	0.518^b^
Site of second surgery (Left/Right)	9/13	11/8	11/9	10/11	12/8	11/9	10/9	0.905^a^
Hypertension (Present/ Absence)	8/14	6/13	10/10	7/14	7/13	9/11	8/11	0.888^a^
Diabetes mellitus (Present/ Absence)	3/19	3/16	4/16	6/15	5/15	6/14	5/14	0.814^c^
Classification of cataracts								1.000^c^
Nuclear	13	10	13	11	14	13	12	
Cortical	3	2	2	3	1	2	2	
Posterior subcapsular	2	2	1	2	1	2	1	
Mixed	4	5	4	5	4	3	4	
Axial length	23 (20–27)	23 (20–27)	23.5(20–26)	23 (20–26)	23 (20–27)	23 (20–26)	24 (20–27)	0.924^b^
Intraocular pressure	15 (11–20)	15 (10–20)	15 (12–19)	15 (11–20)	15 (11–20)	14 (11–20)	15 (11–20)	0.982^b^
Nuclear grading	3.5 (2–5)	4 (2–5)	4 (2–5)	4 (2–5)	4 (2–5)	3 (2–5)	3 (2–5)	0.861^b^
Mean operating room time (min)	18 (13–26)	19 (13–28)	18.5 (13–26)	19 (13–26)	18 (13–26)	18.5 (13–26)	19 (15–25)	0.964^b^
Mean phacoemulsification time (min)	3.5 (2–5)	3 (2–5)	3 (2–6)	3 (2–5)	3 (2–5)	3 (2–5)	4 (2–5)	0.978^b^
MCP-1 (pg/ml)	3799 (2658–4989)	3076 (2271–3676)	3222.5 (2357–3582)	3337 (2488–3792)	3126.5 (2747–3998)	3868 (3214–4618)	3267 (2776–3822)	*< 0.001*^*b*^
Value of anxiety scores								
STAI	8 (6–21)	8 (6–24)	8 (6–24)	8 (6–21)	9 (6–23)	8.5 (6–24)	8 (6–23)	0.960^b^
VAS anxiety	2 (0–7)	2 (0–8)	2 (0–8)	1 (0–8)	2 (0–5)	1.5 (0–8)	1 (0–8)	0.885^b^
VAS pain scores	3 (1–7)	2 (0–6)	2 (0–6)	2 (0–6)	2 (0–7)	3.5 (1–7)	2 (0–5)	*0.001*^*b*^

*P* value of less than 0.050 was considered to indicate a significant difference

*HR* heart rate, *STAI* State–Trait Anxiety Inventory, *VAS* visual analog scale, *MCP-1* monocyte chemoattractant protein-1

*Note*: ^a^X^2^ test; ^b^Wilcoxon test; ^c^Fisher’s exact test

**Fig. 2:**
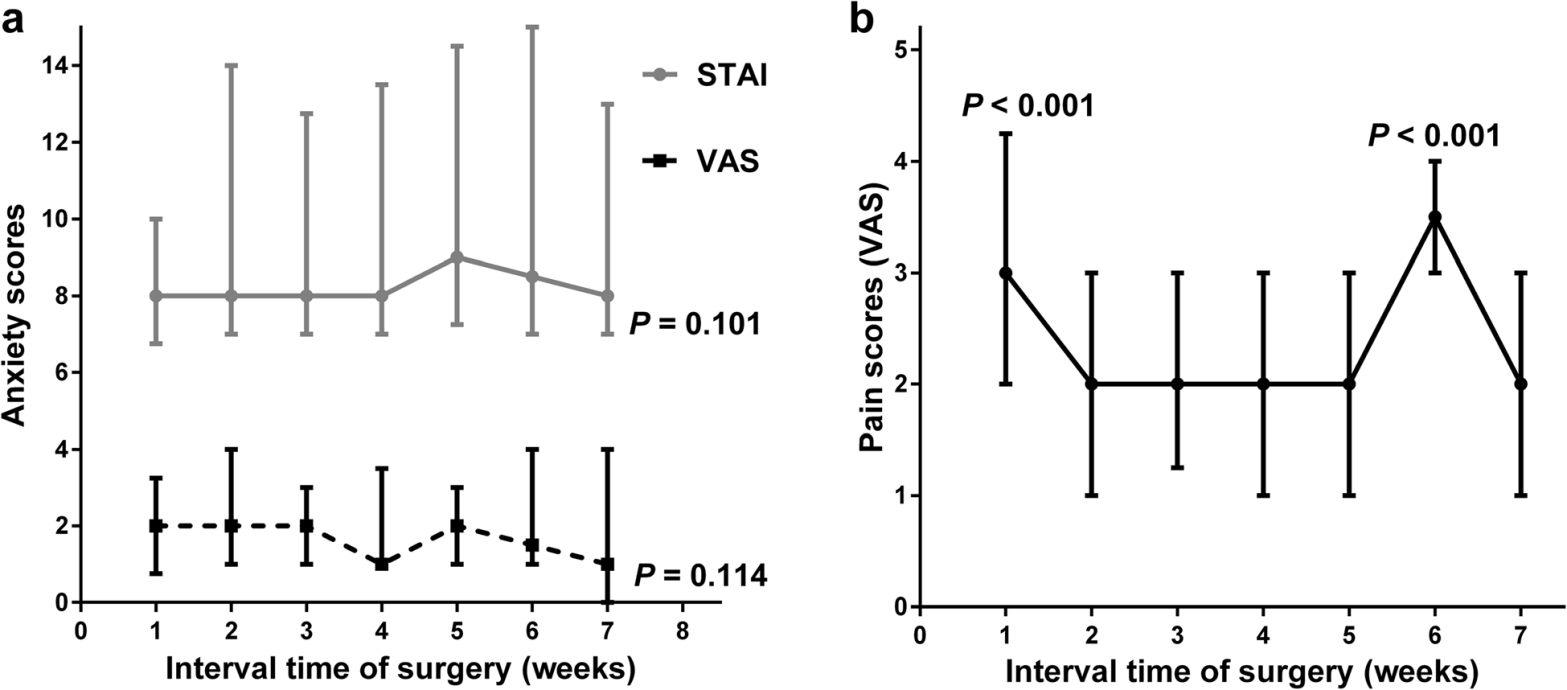
Comparison of anxiety (**a**) and pain score (**b**) (median with range) in seven subgroups established based on the interval between first-eye and second-eye cataract surgery

Anxiety score including STAI and VAS did not statistically different among all the subgroups (Fig. 2a). However, Fig. 2b; Table 3 showed when the interval was at 1-week (median: 3, range: 1–7) or 6-week (median: 3.5, range: 1–7), the VAS pain scores during second-eye surgery were much greater than those when second-eye surgery was performed at other intervals.

### MCP-1 level in aqueous humor

**Fig. 3:**
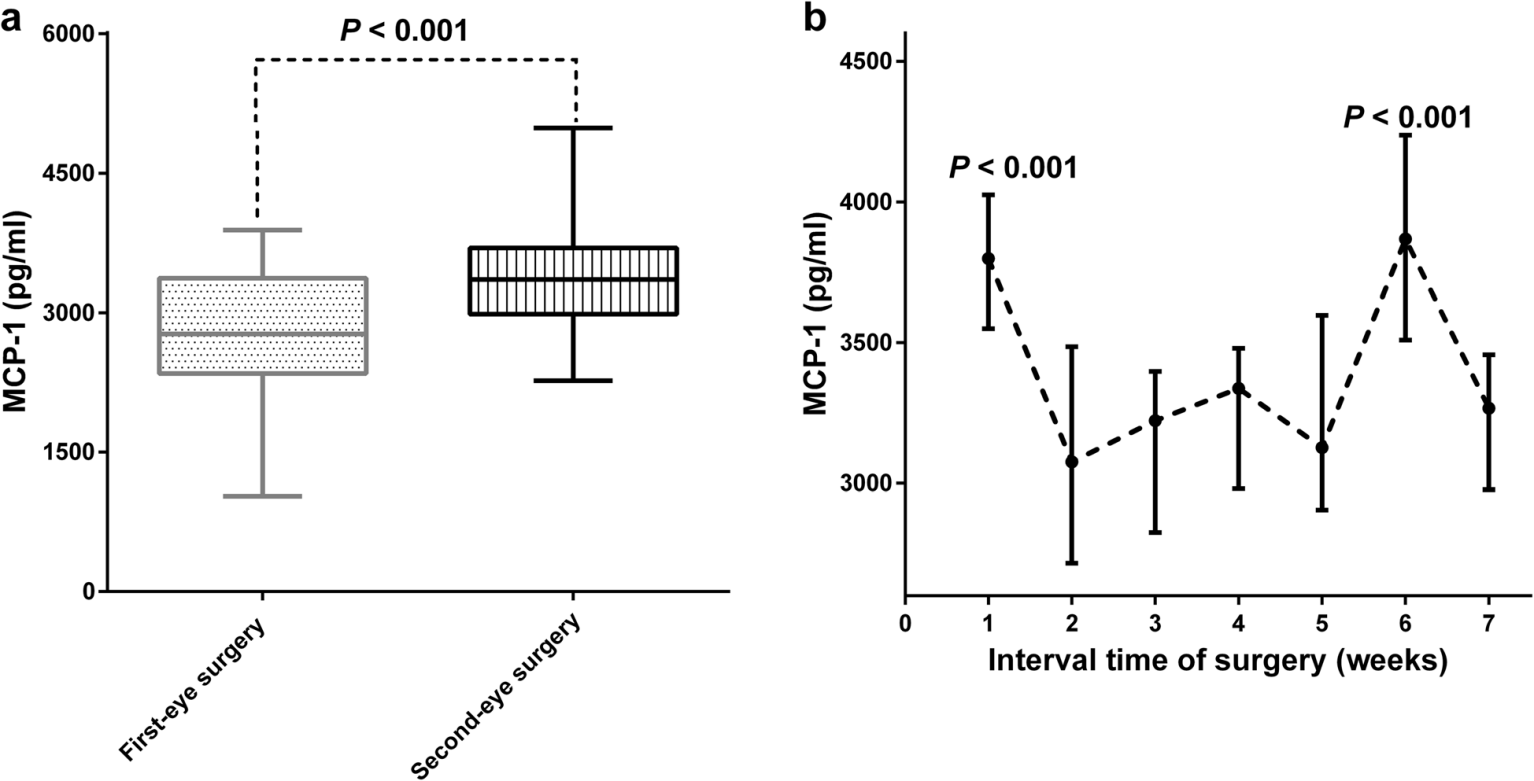
Comparison of MCP- 1levels in aqueous humor between first-surgery and second-surgery (**a**); Comparison of MCP-1 levels in the second eye in seven subgroups based on the interval between first-eye and second-eye cataract surgery (**b**)

Figure 3a indicated that MCP-1 level in aqueous humor was statistically greater in second-eye surgery than that in first-eye surgery (median: 2773, range 1027–3888 vs. medians: 3357, range 2271–4989, *P* < 0.001). What is more, the median MCP-1 level in aqueous humor during first-eye surgery were significantly greater in group 1 (median: 3799, range 2658–4989, *P* < 0.001) and group 6 (median: 3868, range 3214–4618, *P* < 0.001) (Fig. 3b; Table 3).

### Correlation of perioperative pain scores with MCP-1 levels in aqueous humor

**Fig. 4:**
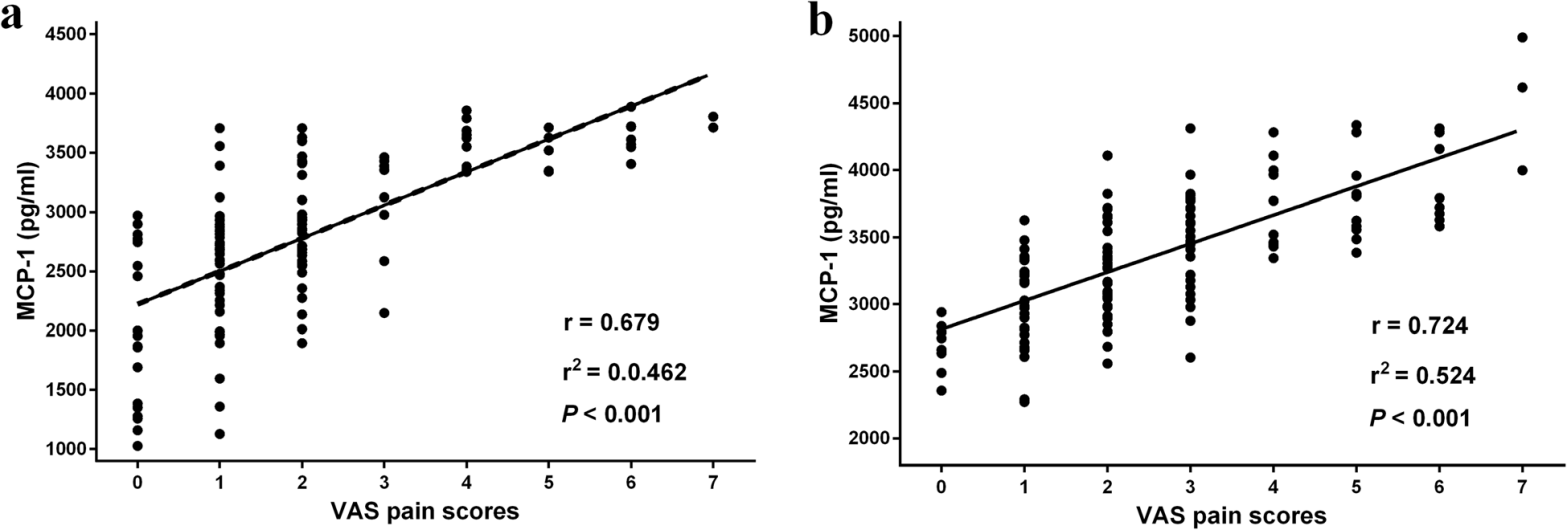
Scatterplots of VAS pain score and MCP-1 levels in aqueous humor during the first-eye (**a**) and second-eye (**b**) surgeries. Pearson’s bivariate correlation analysis was used to identify the relationship

Regarding the correlation of perioperative pain score with MCP-1 levels, results from Fig. 4a showed that perioperative pain was significantly and positively correlated with MCP-1 level in aqueous humor during first-eye surgery r = 0.679, r^2^ = 0.462, *P* < 0.001). Additionally, MCP-1 level across all the subgroups significantly correlated with the VAS pain score in the second-eye cataract surgery, with a correlation coefficient of r = 0.724, r^2^ = 0.524, *P* < 0.001) (Fig. 4b).

## Discussion

Uncomplicated cataract extraction under topical anesthesia is the main surgical approach to treat cataract. However, it is common observation that bilateral cataract patients undergoing sequential phacoemulsification with IOL implantation might experience more painful sensation during second-eye surgery, even though both procedures were performed by a same clinician and under same condition [27]. Perioperative pain not only reduces the patients’ willingness to cooperate but also increases surgical difficulty and reduce patients’ satisfaction [28]. Hence, favorable perioperative pain management is remarkably important. Our results, in agreement with the results from previous studies, demonstrated that patients were prone to experience pain or more severe pain in second eye surgery than in first-eye surgery [14, 15]. Additionally, preoperative heart rate (HR) is useless to predict perioperative perceived pain. When we maximized the comparability by matching baseline and clinical characteristic such as preoperative anxiety between the two groups, similar results were also discovered, which inferred that some factors after the first surgery might affect patients’ perceived pain during second-eye surgery. As we know, psychological factors such as anxiety might influence patient responses to surgery and the degree of perceived pain [29]. Prior studies have found that patients were more relaxed during their second-eye surgery, whereas decreased anxiety might result in increased awareness during the procedure, which might make the sensitivity to pain increased and have a negative influence on experience during the second-surgery [30, 31]. However, our study found no correlation between anxiety and pain score between the first and second surgery, which might be explained by the reason that the influence of anxiety on perceived pain could be minimized by preoperative education.

Then, we further investigated patients’ perceived pain at different intervals between first-eye and second eye cataract surgery. All the recruited patients were subdivided into seven subgroups based on the intervals. Results showed that patients’ VAS pain scores were statistically greater when the interval was at week 1and week 6than those when the second-eye operation was conducted at other intervals, while STAI and VAS anxiety score had no significant difference among subgroups. The findings also confirmed that some factors after a first-surgery might affect patients’ perceived pain during second eye operation [25]. Hence, we conjectured that some dynamic indicators after first-eye surgery might function as predictors of perceived pain during second-eye cataract surgery.

Pain is known to be closely associated with inflammation [32]. Pain-related inflammatory factors might play a critical role in mediating the production of pain [33]. Hence, possible objective mechanism of perceived pain during the second-eye cataract surgery might be identified by comparing the level of pain-related inflammatory factor in aqueous humor. MCP-1, a pain-related cytokine, is predominantly produced by endothelial cells and macrophages and is considered one of the most important potent chemotactic factors for monocytes [34, 35]. Previous studies reported that MCP-1level in aqueous humor was significantly elevated after phacoemulsification [24]. Same with previous research, our study demonstrated that MCP-1 level in aqueous humor was significantly greater during the second-eye surgery than that during first-eye surgery. As a pain-related factor, it has been demonstrated that MCP-1 level contributes to chronic arthritis, fibromyalgia, and glaucoma [36–38]. Therefore, we hypothesized that elevated MCP-1 in aqueous humor might be a key factor causing more intense pain during second-eye surgery compared with first-eye surgery.

Linear regression analysis suggested a strong correlation between MCP-1 level and perioperative VAS pain score during both surgeries, suggesting that MCP-1 level could function as a predictor of patients’ perceived pain. The following reasons might explain this finding. Firstly, phacoemulsification surgery impairs the blood-aqueous barrier and mediates infiltration of inflammatory cell, which could change the microenvironment of aqueous chambers [10, 39]. Those changings might cause MCP-1 maintaining elevated for a long period. Secondly, MCP-1 function as a biomarker of sympathetic eye condition, one of the most well-known being sympathetic ophthalmia [25, 40]. The possible mechanism involved in the pathogenesis of sympathetic ophthalmia is T-lymphocyte mediated delayed hypersensitivity [25, 41]. The downstream effects could be mediated by cytokines including IL-6 and MCP-1 [42, 43]. In turning, those factors could also promote inflammatory mediator secretion [44]. Thirdly, unilateral eye surgery can cause local inflammatory reaction in both eyes [45].

We also discussed the relationship of the interval between both surgeries of perceived pain and MCP-1 level during the second eye surgery. Results showed when the interval was at week 1and week 6, VAS pain score during the second eye surgery was significantly greater than those when surgery was conducted at other intervals. Similarly, the MCP-1 level was also much higher when surgery was performed at week 1 and week 6 intervals. Linear regression analysis suggested MCP-1level strongly correlated with VAS pain score. Hence, dynamic MCP-1 level might function as a predictor of perceived pain in bilateral cataract patients undergoing sequential phacoemulsification eye surgeries. Following reasons might partly explain the results. Firstly, unilateral eye surgical procedure might lead to acute inflammatory response in both eyes. The acute inflammatory response further regulates the production of inflammatory cytokines including MCP-1 [46]. When the second-eye surgery was performed after intervals of 1-week, the MCP-1 in the second-eye remained a stable and high level. Secondly, a delayed inflammatory response involved in MCP-1 level might explain the reason that the VAS pain score was considerably higher in 6-week group [16]. Finally, using non-steroidal drug can dramatically reduce MCP-1 level and relieve perceived pain after sequential second-eye cataract surgery [47, 48].

The findings of the present study should be interpreted in consideration of its possible limitations. First, the study is a retrospective and a single center design with relatively small sized sample, which might have a negative impact on the findings. Besides, relevant mechanism involved in the specific pathway and infiltration of inflammatory cells in the contralateral eye was not investigated in the present study. Therefore, further basic researches need to be performed to identify the detailed mechanism and our findings remain to be confirmed by multicenter prospective clinical studies.

## Conclusions

In conclusion, MCP-1 level in aqueous humor significantly correlated with perceived pain during cataract surgery. Dynamic MCP-1 level could function as predictors of perceived pain during the first and second phacoemulsification eye surgeries in patients with bilateral cataract, which might support clinicians in treatment optimization and clinical decision-making.

## Data Availability

The datasets generated and/or analyzed during the current study are not publicly available due to requirements from the second hospital of Jilin university, but are available from the corresponding author on reasonable request.

## Abbreviations

MCP-1: Monocyte chemoattractant protein-1
IOL: Intraocular lens implantation
STAI: State-Trait Anxiety Inventory
VAS: Visual analog scale
ELISA: Enzyme linked immunosorbent assay
